# Surveillance of mosquito vectors in Southern Sweden for Flaviviruses and Sindbis virus

**DOI:** 10.1080/20008686.2019.1698903

**Published:** 2019-12-05

**Authors:** Jenny C. Hesson, Emma Lundin, Åke Lundkvist, Jan O. Lundström

**Affiliations:** a Department of Medical Biochemistry and Microbiology/Zoonosis Science Center, Uppsala University, Uppsala, Sweden; b Biologisk Myggkontroll, Nedre Dalälven Utvecklings AB, Gysinge, Sweden

**Keywords:** *Culex torrentium*, *Culex pipiens*, pan-flavi PCR, infection rate, West Nile virus, Usutu virus

## Abstract

There are three human pathogenic bird-viruses transmitted by *Culex* mosquitoes in Europe: the alphavirus Sindbis and the flaviviruses West Nile virus and Usutu virus. Cases of Sindbis fever occur in the north while the flaviviruses are reported from southern Europe. In this study, 7933 *Culex pipiens/torrentium* mosquitoes from southern Sweden were screened by RTqPCR for these viruses. None of the mosquitoes were positive for viral RNA. The importance of mosquito species composition is discussed as a potential explanation to the lack of detection of mosquito-borne viruses in southern Sweden. However, continued surveillance of mosquitoes for Flaviviruses would be valuable as an early warning for public health awareness.

## Introduction

In Europe, there are three human pathogenic viruses transmitted from birds, by mosquitoes, to humans: West Nile virus (WNV), Usutu virus (USUV) (Flaviviridae) and Sindbis virus (SINV) (Togaviridae). The transmission of all the three viruses is dependent on mosquitoes from the genus *Culex*. The distribution and importance of Flaviviruses is increasing in Europe, with both WNV and USUV detected from mosquitoes and birds as far north as Germany [1–3], and with human and equid WNV cases as far north as the Czech Republic and Germany [4,5]. USUV was also recently detected in birds in Belgium and in one dead Blackbird in southern Sweden [6,7]. A key question is: how far north is there a risk of Flavivirus transmission to humans?

SINV exemplifies the opposite situation; it is endemic in Finland and central and northern Sweden. In these regions, SINV is often detected in mosquitoes and reports of the disease occur regularly [8–12]. SINV has also been detected in mosquitoes in central and southern Europe, but with a significantly lower infection rate and with no reports of human cases [2,13,14].

## Materials and methods

In this study, mosquitoes were sampled in rural and suburban surroundings of the city of Kristianstad in southern Sweden (56,0387; 14,14,380), where the probability of Flavivirus introduction from the south would be the highest and where SINV have never been reported to cause disease. Sampling was performed every second week from mid-July to mid-September in 2006, 2007 and 2008, using Centers of Disease Control miniature light traps (CDC-traps) baited with carbon dioxide. A cold chain using dry ice was maintained from the time of emptying the traps until storage at −80 degrees Celsius. Mosquitoes were morphologically identified on a chill-table using a stereomicroscope and keys by Becker et al. [15]. All *Cx. pipiens/torrentium* were sorted out, and 1–50 mosquitoes were pooled and used for RNA extraction using Qiagen RNeasy mini kit, following the manufacturer’s instructions (Qiagen, Hilden, Germany). Extracted RNA was then screened for Flaviviruses by a pan-flavi RTqPCR using the QuantiTect® SYBR® Green RT-PCR kit, following the manufacturer’s instructions (Qiagen, Hilden, Germany). The primers used were Flavi all S (5′-TACAACATGATGGGGAARAGAGARAA-3′), Flavi all S2 (5′-TACAACATGATGGGMAAACGYGARAA-3′) and Flavi all AS4 (5′-GTGTCCCAGCCNGCKGTRTCRTC-3′), designed by Patel et al. [16]. The PCR protocol was initially set-up and evaluated by using mosquitoes experimentally infected with WNV. In addition, all samples were screened for SINV by a SINV specific RTqPCR using the Power SYBR™ Green RNA-to-CT™ 1-Step Kit (Thermo Scientific, Vilnius, Lithuania) [13]. This protocol was also quality-tested in earlier studies on field-infected mosquitoes [11].

## Results

The modified pan-flavi RTqPCR could detect 10^3^ PFU/mL WNV and the cut off was set at cycle threshold 38. The number of *Cx. pipiens/torrentium* mosquitoes collected over the years varied considerably, with 107 collected in 2006, 7746 in 2007, and 80 in 2008. In total, 7933 *Cx. pipiens/torrentium* mosquitoes were screened with RTqPCR, and none of the samples were positive for either Flaviviruses or for SINV.

## Discussion

The number of *Cx. pipiens/torrentium* collected in 2007 was extraordinary high for Sweden. However, despite the large sample size, no flaviviruses or SINV were detected. During the years studied, 2006–2008, transmission of WNV was still mainly reported from the Mediterranean and Eastern European countries, such as France and Romania [17], while USUV was transmitted in a few central European countries such as Austria and Hungary [18]. Since then, the reports of both WNV and USUV have increased, indicating a spread of the viruses. However, seroconversion of sentinel chickens to both WNV and USUV was detected on the British Isles already in 2004 [19], so it is possible that both viruses were circulating in larger areas of Europe than detected at the time. Our results indicate that no flaviviruses were circulating in southern Sweden during these years.

The occurrence of human cases usually depends on a high infection rate in the mosquito vector population. This is necessary for transmission to be high enough for spill-over cases from birds, which are the natural vertebrate host of these viruses, to humans, which are considered dead-end hosts. Surveillance of mosquitoes for viruses is therefore often utilized as an early warning system for human epidemics. The lack of detection of SINV in 7746 *Cx. pipiens/torrentium* mosquitoes in southern Sweden in 2007, can be compared to the infection rate in an endemic area in Sweden in 2009 where 14/668 *Cx. pipens/torrentium* were infected with SINV [11]. This shows that vectors in southern Sweden are infected with SINV much more seldom than in central and northern Sweden, which might explain the lack of cases in the southern regions. In addition, an earlier study [20] that focused on species determination of *Cx. pipiens* and *Cx. torrentium* showed that 92% of the adult *Culex* mosquitoes caught in Kristianstad in 2007 were *Cx. pipiens*. In endemic areas further north in Sweden, *Cx. torrentium* is the dominating *Culex* species, comprising over 80% of the *Culex* population [20]. *Cx. torrentium* is also infected with SINV significantly more often than *Cx. pipiens* (infection rates of 36/1000 and 8/1000, respectively) [11]. In central European countries like Germany, *Cx. torrentium* seldom makes up more than 50% of the *Culex* population [21]. This indicates that a high abundance, reaching 80% or more, of *Cx. torrentium* might be necessary for the epidemic spread of SINV to humans to occur.

The use of CDC light traps is not ideal for collecting these *Culex* species. We have previously shown that these traps pose a bias towards catching *Cx. pipiens* over *Cx. torrentium* [12]. Thus, the absence of SINV in this study could be due to the low number of *Cx. torrentium* sampled. However, the infection rate of *Cx. pipiens* in endemic areas can reach 8 infected mosquitoes in 1000 individuals [11], which should have been detected in the collections of over 7000 mosquitoes in this study. Future investigations on the impact of individual vector species abundance on virus transmission should ideally include sampling over a latitudinal gradient using unbiased traps, such as CDC-gravid traps, in combination with virus detection as well as seroprevalence data in the human and avian population.

In this study no Flaviviruses were detected, however, continued surveillance of mosquitoes in southern Sweden for Flaviviruses would be valuable as an early warning for public health awareness.
